# Vertically Aligned Single-Crystalline CoFe_2_O_4_ Nanobrush Architectures with High Magnetization and Tailored Magnetic Anisotropy

**DOI:** 10.3390/nano10030472

**Published:** 2020-03-05

**Authors:** Lisha Fan, Xiang Gao, Thomas O. Farmer, Dongkyu Lee, Er-Jia Guo, Sai Mu, Kai Wang, Michael R. Fitzsimmons, Matthew F. Chisholm, Thomas Z. Ward, Gyula Eres, Ho Nyung Lee

**Affiliations:** 1 Oak Ridge National Laboratory, Oak Ridge, TN 37831, USA; lfan@zjut.edu.cn (L.F.); g.xiang@outlook.com (X.G.); thomas.farmer27@hotmail.co.uk (T.O.F.); dongkyu@cec.sc.edu (D.L.); ejguo@iphy.ac.cn (E.-J.G.); sai.mu1986321@gmail.com (S.M.); wangkai.mse@outlook.com (K.W.); fitzsimmonsm@ornl.gov (M.R.F.); chisholmmf@ornl.gov (M.F.C.); wardtz@ornl.gov (T.Z.W.); eresg@ornl.gov (G.E.); 2 College of Mechanical Engineering, Zhejiang University of Technology, Hangzhou 310023, Zhejiang, China; 3 Department of Physics and Astronomy, University of Tennessee, Knoxville, TN 37996, USA

**Keywords:** cobalt ferrite, nanobrush, pulsed laser epitaxy, vertically aligned, single crystalline, magnetization, tailored anisotropy

## Abstract

Micrometer-tall vertically aligned single-crystalline CoFe_2_O_4_ nanobrush architectures with extraordinarily large aspect ratio have been achieved by the precise control of a kinetic and thermodynamic non-equilibrium pulsed laser epitaxy process. Direct observations by scanning transmission electron microscopy reveal that the nanobrush crystal is mostly defect-free by nature, and epitaxially connected to the substrate through a continuous 2D interface layer. In contrast, periodic dislocations and lattice defects such as anti-phase boundaries and twin boundaries are frequently observed in the 2D interface layer, suggesting that interface misfit strain relaxation under a non-equilibrium growth condition plays a critical role in the self-assembly of such artificial architectures. Magnetic property measurements have found that the nanobrushes exhibit a saturation magnetization value of 6.16 μB/f.u., which is much higher than the bulk value. The discovery not only enables insights into an effective route for fabricating unconventional high-quality nanostructures, but also demonstrates a novel magnetic architecture with potential applications in nanomagnetic devices.

## 1. Introduction

Metal oxides, particularly complex transition metal oxides, are highly desired for their wide range of novel properties and potential applications [1]. Exploration of new fabrication routes for unconventional metal oxide nanostructures has attracted increasing attention due to their novel size-dependent properties, which opens up new avenues of functionalities in diverse fields including electronics, photonics, sensors, catalysis, energy harvesting, and information storage [2,3,4]. In particular, vertically ordered single-crystalline nanostructures have significant advantages over conventional planar films due to the enhanced vertical surface area, control over the shape anisotropy, and efficient vertical transport of electrons and optical excitations, which are critical to the function and integration of components at the nanoscale [5,6,7,8]. The ability to fabricate such nanostructures is essential in modern science and technology; to whit, understanding their growth mechanisms at atomic level is imperative.

In our previous studies, we demonstrated the versatility of pulsed laser epitaxy (PLE) to tailor a variety of isolated vertically aligned single-crystalline nanostructures by balancing the kinetic and thermodynamic non-equilibrium PLE processes [9,10]. This balance provides a simple but intriguing strategy for constructing unconventional vertically aligned single-crystalline binary oxide nanostructures in terms of material diversity including CeO_2_, Y_2_O_3_, and TiO_2_ [9,10]. In this work, we demonstrate the application of the technique to the synthesis of a ternary ferrimagnetic spinel oxide, CoFe_2_O_4_ (CFO) nanobrush. In the past few decades, CFO has been intensively investigated due to intriguing potential applications in spintronics, for example, as one component in multi-ferroic heterstructure [11,12], or as a spin filter tunneling barrier [13,14], etc. The magnetic properties (saturation moment, magnetic ground states, anisotropy) of CFO is a strongly structure dependent, and thus can be manipulated by epitaxial strain through the magnetostriction effect [15,16,17,18,19], and the extent of the inversion of the spinel structure [20,21,22]. The fabrication of high-density vertically ordered arrays of single crystalline CFO nanomagnets is of fundamental importance to the emerging information technologies such as ultrahigh density storage devices, magnetic random access memory devices, and logic devices [23,24,25,26].

In this work, micrometer-tall vertically aligned single-crystalline CFO “nanobrushes” were fabricated within a small window of kinetic growth parameters. Diffusion limited aggregation (DLA) is a process whereby the incoming adatoms move in a random path to form clusters, the growth front roughness thus highly depends on the surface diffusion kinetics. The formation of CFO nanobrushes occurs due to a delicate balance between thermodynamic surface equilibration and kinetic DLA. It was found that the interface misfit strain relaxation plays a critical role in the construction of the unique nanobrush structure. The nanobrushes exhibit an abnormally high saturation magnetization value of 6.16 μB/f.u., associated with a thickness-dependent magnetic anisotropy.

## 2. Materials and Methods 

CFO samples were epitaxially grown on (001) SrTiO_3_ (STO) substrates by a home-made PLE system (Oak Ridge, TN, USA). The STO substrates were pretreated by buffered hydrofluoric acid (HF) for 30 s and thermally treated at 1100 °C in air for 1.5 h to ensure TiO_2_-terminated surfaces. A KrF excimer laser (pulse duration: 25 ns, laser fluence: 1.2–2.1 J/cm^2^, laser repetition rate: 15 Hz) was used to ablate a stoichiometric CFO ceramic target for deposition at a substrate temperature, *T*, in a range of 400–700 °C in an oxygen partial pressure, *p*(O_2_), with a range of 0.1–1 Torr. The typical deposition rate was 0.24 Å per pulse. 2D continuous CFO films were prepared at a *p*(O_2_) of 10 mTorr, a substrate temperature of 700 °C, and a laser fluence of 0.8 J/cm^2^.

The structural quality of the CFO samples was examined by x-ray diffraction (XRD) using a four-circle high-resolution x-ray diffractometer (X’Pert Pro, PANalytical, Almelo, Netherlands; Cu K α_1_ radiation). The surface morphology of the samples was characterized by scanning electron microscopy (SEM). The macroscopic magnetic properties were measured with magnetic field applied along both in-plane and out-of-plane directions using a superconducting quantum interference device (SQUID, Quantum Design, San Deigo, CA, USA).

Cross-sectional specimens oriented along the [110] STO direction for scanning transmission electron microscopy (STEM) analysis were prepared using ion milling after mechanical thinning and precision polishing (using water-free abrasive). High-angle annular dark-field STEM (HAADF-STEM) was carried out in a Nion UltraSTEM microscope operated at 100 keV (Nion Co., Kirkland, WA, USA). The microscope was equipped with a cold field-emission gun and a corrector of third- and fifth-order aberrations for sub-Ångstrom resolution. An inner detector semi-angle of ~78 mrad was used for HAADF imaging. The convergence semi-angle for the electron probe was set to 30 mrad.

## 3. Results

### 3.1. Self-Assembly of the CoFe_2_O_4_ Nanobrushes

**Figure 1:**
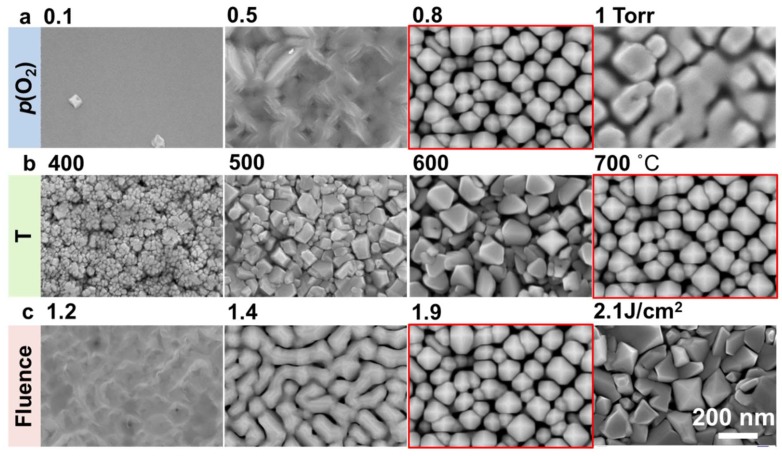
Plan-view SEM images of surface morphologies of CoFe_2_O_4_ samples prepared at different (**a**) oxygen pressure (*p*(O_2_)), (**b**) substrate temperatures (*T*), and (**c**) laser fluences (*J*). Other growth conditions for the samples shown in (**a**) are *T* = 700 °C and *J* = 1.9 J/cm^2^; for (**b**), *p*(O_2_) = 0.8 Torr and *J* = 1.9 J/cm^2^; for (**c**), *p*(O_2_) = 0.8 Torr and *T* = 700 °C.

As demonstrated in our previous work [9,10], self-assembly of highly epitaxial, vertically aligned, single-crystalline oxide nanostructures was achieved by controlling adatom surface equilibration and DLA far from thermodynamic equilibrium. In a similar manner, the development of the surface morphologies as a function of kinetic growth parameters (substrate temperature, reactive gas pressure, and laser fluence) was fully mapped out through the plan-view SEM images of the CFO film surfaces in Figure 1. As shown from the XRD *θ*–2*θ* scans in Figure S1, all films were epitaxially grown on STO (001) with only the (00*l)* series of the CFO peaks, except for the one prepared at 1 Torr that showed weak impurity peaks.

The CFO sample prepared at 700 °C, 0.8 Torr, and 1.9 J/cm^2^ shows a distinct loosely packed surface morphology consisting of well-defined pyramid-like heads. It is noted that this unique porous structure formed at an extremely high *p*(O_2_) value, which is not normally used for conventional continuous 2D film epitaxy. The high reactive gas pressure is mainly responsible for the small kinetic energy of the arriving adatoms, giving rise to DLA and growth front roughness [5]. Furthermore, a high substrate temperature is essential to form the well-defined pyramids as opposed to the randomly shaped crystals observed at low temperatures. The large thermal energy provided by the hot substrate drives the adatoms to locally arrange into the lattice correctly, resulting in the growth of ordered epitaxial structures [27]. Relatively high laser fluence is also critical to the formation of this architecture, because it prevents adatoms from bouncing off and eliminating the long-distance diffusion of the adatoms by quickly covering them with the new incoming atoms [28,29,30,31]. This matches our previous observations with CeO_2_, Y_2_O_3_, and TiO_2_ systems [9,10], in which balancing the DLA and surface equilibrium allows spontaneous breakdown of the layer-by-layer growth while ensuring high crystallinity.

**Figure 2:**
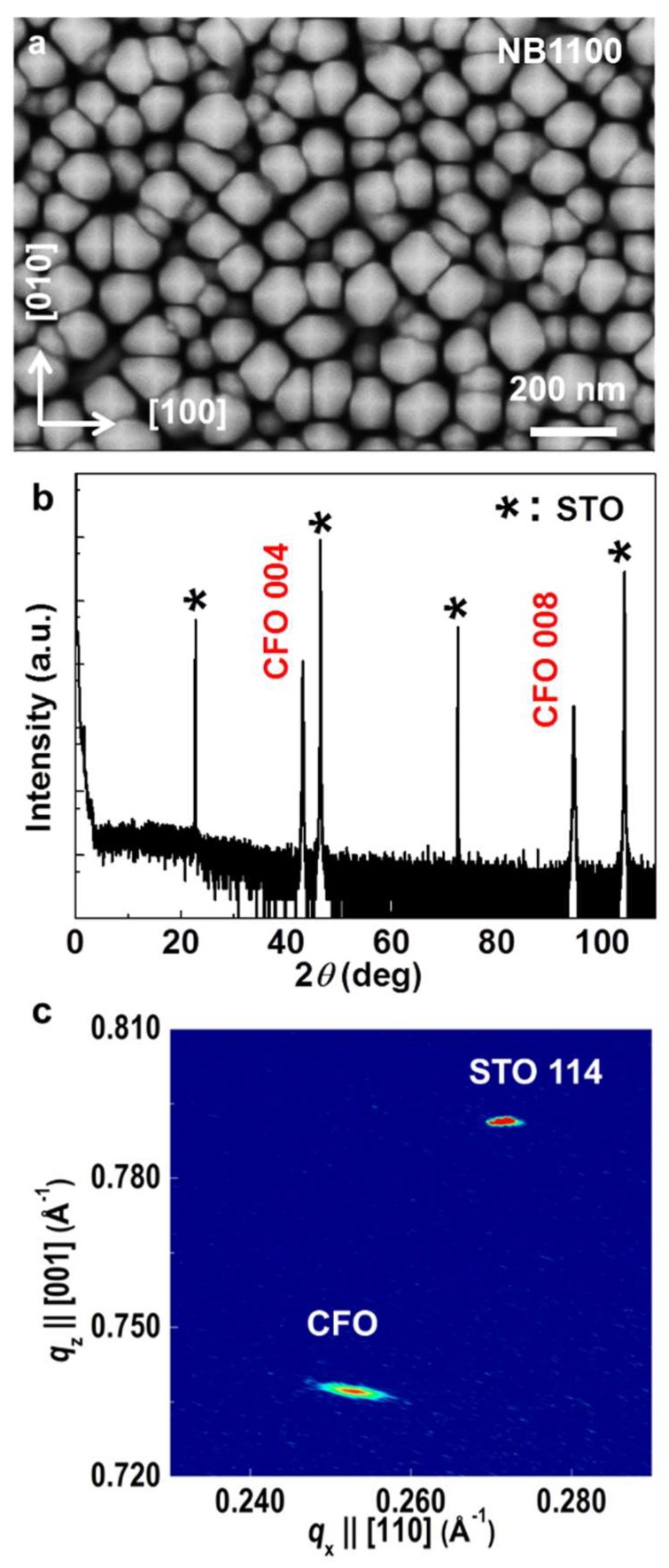
(**a**) A plan-view SEM image with pyramid-shaped tips clearly seen from a micrometer-tall CoFe_2_O_4_ nanobrush sample (NB1100), (**b**) a high-resolution XRD *θ*–2*θ* scan, and (**c**) an x-ray reciprocal space map around the 114-reflection of SrTiO_3_ (STO) of sample NB1100 showing the biaxial strain of the nanobrush is highly relaxed. * denotes the STO {00l} substrate peaks.

From the SEM image of the CFO sample (Figure 2a), the alignment of the pyramid-shaped heads was consistent along the <100> directions. Each individual pyramid has a size ranging from 80 to 150 nm in width. No impurity phases were detected in the XRD *θ*–2*θ* scan (Figure 2b) of the (001)-oriented CFO sample. Based on the Bragg diffraction theory, the CFO sample has a lattice constant of 8.386 ± 0.002 Å, which is located in between the reported values in JPCDS Card# 3-384 (8.377(7) Å) and the Card# 22-1086 (8.3919 Å) for stoichiometric CFO and comparable to the reported value of 8.39 Å in [32] for a cobalt ferrite bulk, which is slightly rich in Co with a Co:Fe ratio of 1:1.98. According to this, the CFO sample is near stoichiometric. The crystallinity of the CFO was evaluated by rocking curve measurements (Figure S2a). The typical full width at half-maximum (FWHM) of the 004 reflection was ~0.38°, revealing good epitaxial quality. The in-plane *ϕ* scans (Figure S2b) of the CFO 101 and STO 101 reflections at *ψ* = 45° revealed a clear four-fold symmetry, demonstrating the cube-on-cube registration relationship between the CFO sample and the STO substrate. X-ray reciprocal space mapping (RSM) near the off-specular 114-reflection of the STO substrate was performed in order to check the strain state as displayed in Figure 2c. The CFO sample shows both in-plane 7.48% and out-of-plane 7.28% lattice strain relaxations. CFO and STO have in-plane lattice constants of 8.39 Å and 3.92 Å, respectively, leading to a lattice misfit value of 7.4%. The fact that the strain relaxation is close to the lattice mismatch between bulk CFO and STO suggests that both in-plane and out-of-plane lattice strains are fully relaxed in the CFO sample.

### 3.2. Microstructure of the CoFe_2_O_4_ Nanobrushes

**Figure 3:**
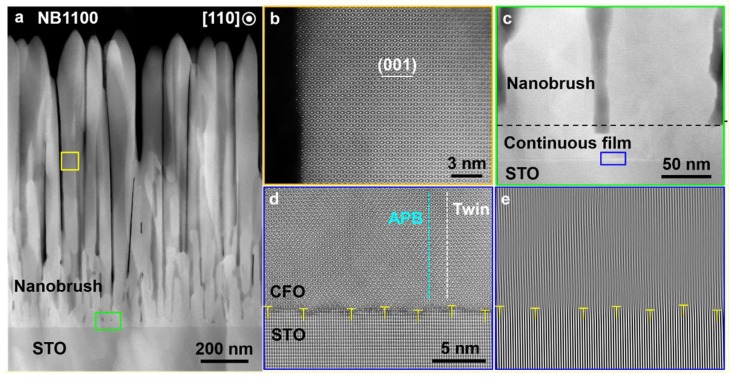
(**a**) A low-magnification cross-sectional high-angle annular dark-field (HAADF) image of Sample NB1100. High-resolution HAADF images of the regions marked by a yellow and a green rectangle in (**a**): (**b**) the nanobrush sidewall and (**c**) magnified interface microstructure. (**d**) High-resolution HAADF observation of a blue rectangle region in (**c**), showing the film/interface atom structure and (**e**) its fast-Fourier transformation (FFT) map.

Cross-sectional STEM imaging showed the sample was composed of unique vertically aligned “nanobrushes” with a thickness of 1100 nm (Sample NB1100), as shown in Figure 3a. Sample NB1100 exhibits a two-layer structure: a dense thin interfacial layer connecting directly with the substrate, above which vertically aligned crystalline nanobrushes with a micrometer length were grown. The nanobrush diameter ranged from 80 to 120 nm and neighboring nanobrushes were separated by voids, forming a dense brush architecture.

To shed light on the formation of the nanobrushes, HAADF-STEM observation of Sample NB1100 along the CFO [110] direction was performed. The results showed that the single crystal nanobrushes were free from any obvious lattice defects (as shown in Figure 3b) and epitaxially grown. Typically, impurity phases were observed in epitaxial CFO films that lose coherence with the underlying substrate. We did not observe any impurity phase or even defects in the STEM nor XRD with the nanobrush structures that were not coherent with the substrate. Figure 3c clearly shows a continuous layer with an average thickness of 50 nm existing between the nanobrushes and the substrate. The interface displays a clear, albeit a less-abrupt interfacial region, between the dense interface layer and the substrate with slight intermix of the CFO lattice with the STO lattice no more than two unit cells (thin dark region of Figure 3d). The intermix can be attributed to damage caused by the incoming atoms present at the required high energy synthesis conditions.

As revealed by the fast-Fourier transformation (FFT) map in Figure 3e, periodic dislocation arrays were formed at the CFO/STO interface. The average periodicity was about 4.0 nm. Compared to the standard lattice misfit of 7.4% at the CFO/STO interface, the result suggests nearly full lattice misfit compensation by forming the interfacial dislocations. The results indicate that the thin CFO layer epitaxially grown on the substrate was initially under compressive stress due to the large lattice misfit, which favors the formation of interfacial dislocations to compensate for the misfit strain once the layer reaches the critical thickness [33]. The near-interface dense layer consists of nanodomains with anti-phase boundaries (APB) and twin boundaries formed in-between as shown in Figure 3d. The defects in the near-interface dense layer were not found in the nanobrush region. The areas between neighboring defects are energetically favorable for the epitaxial growth, leading to a rough growth front with the dimples corresponding to the defective area, whereas the peaks correspond to the defect-free areas [8]. A shadowing effect that reduces the probability of deposition in dimples will cause this growth front roughness to develop into extended nanobrushes [27].

### 3.3. Magnetization of the CoFe_2_O_4_ Nanobrushes

**Figure 4:**
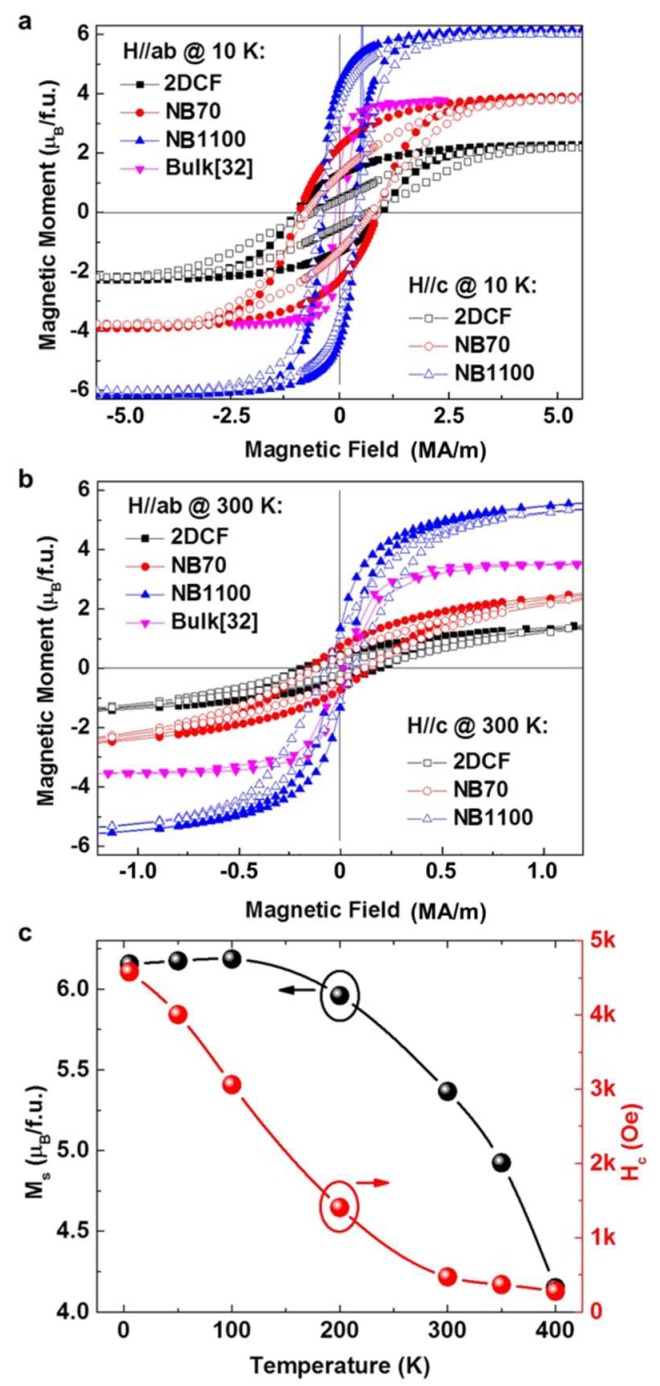
The M–H curves of Sample NB1100, a 70 nm tall nanobrush sample (NB70), and a 180 nm thick 2D continuous film (2DCF) under in-plane (H//ab) and out-of-plane (H//c) magnetic fields measured at temperatures of (**a**) 10 K and (**b**) 300 K, respectively. The M–H curve of the CoFe_2_O_4_ bulk was retrieved from [32] for comparison. (**c**) The coercive field (*H_c_*) and the saturated magnetization (*M_s_*) of the NB1100 plotted as a function of temperature.

Figure 4a,b compare the in-plane and out-of-plane M–H hysteresis loops of the 1100 nm tall nanobrush Sample NB1100, a 70 nm tall nanobrush sample (NB70), and a 180 nm thick 2D continuous film (2DCF). The morphology and XRD characterization of these films are shown in Figure S3. The magnetic parameters extracted from the loops are listed in Table 1. As shown in Figure S3, NB1100 and NB70 exhibited mesoscale porous structures with a mean column width of 83.67 nm and 28.53 nm, respectively. Estimation of the volume fraction of CFO nanobrushes in the NB1100 is critical for accurate determination of its saturation magnetization value. The detailed estimation process of the volume fraction of CFO nanobrushes equal to 63 ± 10% is provided in the Supplementary Materials and Figure S4. Using this value for the volume fraction of nanobrushes, the NB1100 had an in-plane saturation magnetization value of *M_s_* = 6.16 ± 0.7 μ_B_/f.u. at 10 K. W. H. Wang reported the flux growth of bulk single crystal CFO with the 004 reflection FWHM of 0.15° and a saturation magnetization of 3.65 μB/f.u. [32]. The measured saturation magnetization of the NB1100 was almost three times that of the 2DCF and much higher than the reported bulk value [32]. The large saturation magnetization reduced slightly at 300 K. As shown in Figure S3, the NB70 and the 2DCF had much larger widths of the 004 Bragg reflections (e.g., full width at half maximum values 0.73° for the NB70 and 0.83° for the 2DCF) than the NB1100, 0.38°, suggesting lower crystalline quality (e.g., due to defects) compared to NB1100. The high saturation magnetization exhibited by NB1100 can be attributed to the high crystallinity of the nanobrushes and a possible extremely low inversion ratio, *x,* tending toward a normal spinel (all Fe^3+^ on octahedral sites and all Co^2+^ on tetrahedral sites). At thermodynamic equilibrium, CFO should possess an inverted spinel structure where half of the Fe^+3^ are on the tetrahedral sites and the remaining Fe^3+^ plus the Co^2+^ are on the octahedral sites. As the magnetic moments on the tetrahedral and octahedral lattices are in opposition, the CFO magnetization is strongly dependent on the degree of spinel inversion. Density functional theory calculations suggest the magnetization per formula unit of CFO varies from 3 μ_B_ in a fully inverted spinel structure where the magnetic moments of Fe^3+^ on the tetrahedral sites and the octahedral site are completed, compensated up to 7 μ_B_ as the inversion ratio decreases toward a normal spinal structure where all Fe^3+^ is on the octahedral sites and all Co^2+^ is on the tetrahedral sites [20,21,22]. The spinel inversion of CFO is highly sensitive to the growth conditions, where experimental values ranging from *x* = 0.62 to *x* = 0.93 have been reported [34,35]. W. H. Wang did not provide information on the inversion ratio of the bulk single crystal CFO, but indicated the CFO was not completely inverse [32]. The high saturation moment of 6.16 μ_B_/f.u. may suggest that NB1100 has a partial inversion with a ratio below 0.5. Functionally, such a high magnetization observed in the CFO nanobrush architecture is highly desirable.

The coercivity of different forms of CFO samples under both in-plane and out-of-plane magnetic field directions at 10 K and 300 K are listed in Table 1. Based on the Stoner–Wohlfarth model, coercivity comes from a combined effect of the anisotropy field(~*K*/*M_s_*, *K* is anisotropy constant) and the shape-anisotropy field (~(1-3*D*)*M*_s_, *D* is a demagnetizing factor, *D* = 0 for long cylinder structures) [36]. A greater anisotropy gives rise to a larger coercivity. The *H*_c_ of bulk CFO are small at both 10 K and 300 K [32]. This could be attributed to the fact that cubic structure has weak anisotropy. As the NB1100 has a very large aspect ratio (length of each nanobrush divided by its width) of 9.2 and a higher *M*_s_, we expected a more pronounced contribution to *H*_c_ from the shape-anisotropy field as compared to 2DCF and NB70. However, at 10 K, larger *H*_c_ was observed in 2DCF and NB70 than that in NB1100, which indicates a dominant contribution from the anisotropy field in 2DCF and NB70. In addition, we saw an in-plane *H_c_* (H//ab) marginally larger than out-of-plane *H_c_* (H//c) for the NB70 and 2DCF. However, the reverse was the case for the NB1100. The NB110 was fully relaxed as shown in the reciprocal space mapping analysis in Figure 2c and CFO had a large magnetostrictive constant, thus we attribute the difference to strain in the NB70 and 2DCF and lacking in the NB1100, and the ease with which magnetic response can be manipulated through symmetry change [37]. This is reasonable when considering the extremely large magnetostrictive constant of CFO [38]. From this, it is evident that control over the nanobrush height provides a mechanism for tailoring the magnetic anisotropy. A similar thickness dependent anisotropy has been observed in BFO/CFO nanocomposites [39]. 

Figure 4c shows the temperature-dependent *M_s_*(*T*) and *H_c_*(*T*) in the NB1100. While the *M_s_(T)* only reduces by 32.6% up to 300 *K*, the *H_c_* is strongly temperature dependent and a pronounced reduction is observed. According to the Callen–Callen rule [40], the anisotropy constant is linked to the magnetization via power laws: $K\left(T\right)=K\left(0\right){\left(\frac{M_{s}\left(T\right)}{M_{s}\left(0\right)}\right)}^{n}$, where the *m*^th^ order anisotropy constant obeys *n = m(m + 1)/2*. The temperature dependent anisotropy of Fe and Co metals are well described by this model [36]. For a cubic structure as in CFO, a fourth order anisotropy is expected and therefore *n = 10*. However, from the scaling as shown in Figure S5, we observed a much faster *H_c_* reduction than *n* = 10 in *M_s_*(T)*^n^*. The failure of the Callen–Callen model for this nanobrush system may be ascribed to the fact that the Callen–Callen model merely bridges the anisotropy to the net magnetization while neglecting the effect of different exchange fields on different sublattices. Since CFO is an uncompensated ferrimagnet [36], which involves multiple magnetic sublattices, the Callen–Callen model may be inadequate to describe the subtle temperature dependent exchange field on different sublattices, resulting in an error of the anisotropy at finite temperatures.

## 4. Discussion

A simple but novel synthesis strategy that can be applied extensively in diverse material systems for unconventional vertically aligned nanostructure fabrication was developed. Vertically aligned single-crystalline CFO nanobrushes were epitaxially grown on STO substrates by PLE. Thermodynamically and kinetically balancing DLA and surface equilibration through kinetic growth parameter control provides a route to design epitaxially grown self-assembled nanostructures. The magnetic properties are highly sensitive to both the morphology and brush length. It was found that the nanobrushes exhibited a very high saturation magnetization and a length-dependent magnetic anisotropy. This work provides new insight into the controllable synthesis of vertically aligned nanostructures and demonstrates a novel magnetic structure suitable for applications in nanomagnetic devices.

## Figures and Tables

**Table 1:** Magnetic parameters (saturation magnetization *M*_s_, coercivity *H*_c_) of the CoFe_2_O_4_ samples.

T (K)	Sample	M_s__H//ab (μ_B_/f.u.)	M_s__H//c (μ_B_/f.u.)	H_c__H//ab (MA/m)	H_c__H//c (MA/m)
10	2DCF	2.27	2.19	0.99	0.65
	NB70	3.89	3.85	0.90	0.75
	NB1100	6.16 ± 0.71	6.00 ± 0.63	0.37	0.42
	Bulk [32]	3.65	-	0.10	-
300	2DCF	1.83	1.87	0.19	0.11
	NB70	3.13	3.17	0.15	0.10
	NB1100	5.90 ± 0.61	5.64 ± 0.59	0.05	0.07
	Bulk [32]	3.57	-	0.02	-

## Supplementary Materials

The following are available online at https://www.mdpi.com/2079-4991/10/3/472/s1, Figure S1: XRD scans of the CFO samples prepared as functions of kinetic growth parameters; Figure S2: The crystallinity of Sample NB1100 evaluated by XRD characterization (rocking curve and in-plane *ϕ* scan); Figure S3: Morphology and structural characterization of NB1100, NB70, and a 2D continuous film; Figure S4: Estimation of the volume fraction of Sample NB1100; and Figure S5: Comparison of the normalized *H_c_*(T) curve with *M_s_*^10^(T) curve according to the Callen–Callen rule.

## References

[1] Srivastava A.K. (2014). Oxide Nanostructures: Growth, Microstructures and Properties.

[2] Xia Y., Yang P., Sun Y., Wu Y., Mayers B., Gates B., Yin Y., Kim F., Yan H. (2003). One-Dimensional Nanostructures: Synthesis, Characterization, and Applications. Adv. Mater..

[3] Rorvik P.M., Grande T., Einarsrud M.A. (2011). One-Dimensional Nanostructures of Ferroelectric Perovskites. Adv. Mater..

[4] Xu S., Wang Z.L. (2011). One-dimensional ZnO nanostructures: Solution growth and functional properties. Nano Res..

[5] Infortuna A., H arvey A.S., Gauckler L.J. (2008). Microstructures of CGO and YSZ Thin Films by Pulsed Laser Deposition. Adv. Funct. Mater..

[6] Zhou Y., Park C.S., Wu C.H., Maurya D., Murayama M., Kumar A., Katiyar R.S., Priya S. (2013). Microstructure and surface morphology evolution of pulsed laser deposited piezoelectric BaTiO3 films. J. Mater. Chem. C.

[7] Jiang J., Henry L.L., Gnansekar K.I., Chen C., Meletis E.I. (2004). Self-Assembly of Highly Epitaxial (La,Sr)MnO3 Nanorods on (001) LaAlO3. Nano Lett..

[8] Zhang K., Dai J., Zhu X., Zhu X., Zou X., Zhang P., Hu L., Lu W., Song W., Sheng Z. (2016). Vertical La_0.7_Ca_0.3_MnO_3_ nanorods tailored by high magnetic field assisted pulsed laser deposition. Sci. Rep..

[9] Fan L., Gao X., Lee D.K., Guo E.J., Lee S.B., Snijders P.C., Ward T.Z., Chisholm M.F., Eres G., Lee H.N. (2017). Kinetic controlled fabrication of single-crystalline TiO_2_ nanobrush architecture. Adv. Sci..

[10] Lee D.K., Gao X., Fan L., Guo E.J., Farmer T.O., Heller W.T., Ward T.Z., Eres G., Fitzsimmons M.R., Chisholm M.F. (2017). Non-equilibrium synthesis of highly porous single-crystalline oxide nanostructures. Adv. Mater. Interfaces.

[11] Zheng H., Wang J., Lofland S.E., Ma Z., Mahaddes-Ardabili L., Zhao T., Salamanca-Riba L., Shinde S.R., Ogale S.B., Bai F. (2004). Multiferroic BaTiO_3_-CoFe_2_O_4_ nanostructures. Science.

[12] Zavaliche F., Zheng H., Mohaddes-Ardabili L., Yang S.Y., Zhan Q., Shafer P., Reilly E., Chopdekar R., Jia Y., Wright P. (2005). Electric Field-Induced Magnetization Switching in Epitaxial Columnar Nanostructures. Nano Lett..

[13] Chapline M.G., Wang S.X. (2006). Room-temperature spin filerting in a CoFe_2_O_4_/MgAl_2_O_4_/Fe_3_O_4_ magnetic tunnel barrier. Phys. Rev. B.

[14] Ramos A.V., Santos T.S., Miao G.X., Guittet M.-J., Moussy J.-B., Moodera J.S. (2008). Influence of oxidation on the spin-filtering properties of CoFe_2_O_4_ and the resultant spin polarization. Phys. Rev. B.

[15] Fritsch D., Ederer C. (2010). Epitaxial strain effects in the spinel ferrites CoFe_2_O_4_ and NiFe_2_O_4_ from first principles. Phys. Rev. B.

[16] Zhou S., Potzger K., Xu Q., Keupper K., Talut G., Marko D., Mucklich A., Helm M., Fassbender J., Arenholz E. (2009). Spinel ferrite nanocrystals embedded inside ZnO: Magnetic, electronic, and magnetotransport properties. Phys. Rev. B.

[17] Lisfi A., Williams C.M. (2003). Magnetic anisotropy and domain structure in epitaxial CoFe_2_O_4_ thin films. J. Appl. Phys..

[18] Park J.H., Lee J.H., Kim M.G., Jeong Y.K., Oak M.A., Jang H.M., Choi H.J., Scott J.F. (2010). In-plane strain control of the magnetic remanence and cation-charge redistribution in CoFe_2_O_4_ thin film grown on a piezoelectric substrate. Phys. Rev. B.

[19] Ma J.X., Mazumdar D., Kim G., Sato H., Bao N.Z., Gupta A. (2010). A robust approach for the growth of epitaxial spinel ferrite films. J. Appl. Phys..

[20] Walsh A., Wei S.H., Yan Y., Al-Jassim M.M., Turner J.A. (2007). Structural, magnetic, and electronic properties of the Co-Fe-Al oxide spinel system: Density-functional theory calculations. Phys. Rev. B.

[21] Szotek Z., Temmerman W.M., Kodderitzsch D., Svane A., Petit L., Winter H. (2006). Electronic structures of normal and inverse spinel ferrite from first principles. Phys. Rev. B.

[22] Hou Y.H., Zhao Y.J., Liu Z.W., Yu H.Y., Zhong X.C., Qiu W.Q., Zeng D.C., Wen L.S. (2010). Structural, electronic and magnetic properties of partially inverse spinel CoFe_2_O_4_: A first principles study. J. Phys. D Appl. Phys..

[23] Wang Z., Viswan R., Hu B., Harris V.G., Li J.F., Viehland D. (2012). Tunable magnetic anisotropy of CoFe_2_O_4_ nanopillar arrays released from BiFeO_3_ matrix. Phys. Status Solidi RRL.

[24] Gao X., Liu L., Birajdar B., Ziese M., Lee W., Alexe M., Hesse D. (2009). High-density periodically ordered magnetic cobalt ferrite nanodot arrays by template-assisted pulsed laser deposition. Adv. Funct. Mater..

[25] Guo E.J., Herklotz A., Kehlberger A., Cramer J., Jakob G., Klaui M. (2016). Thermal generation of spin current in epitaxial CoFe_2_O_4_ thin films. Appl. Phys. Lett..

[26] Mathew D.S., Juang R.S. (2007). An overview of the structure and magnetism of spinel ferrite. Chem. Eng. J..

[27] Pelliccione M., Lu T.M. (2008). Evolution of Thin Film Morphology.

[28] Thornton J.A. (1977). High rate thick film growth. Ann. Rev. Mater..

[29] Muller K.H. (1985). Dependence of thin-film microstructure on deposition rate by means of a computer simulation. J. Appl. Phys..

[30] Yang Y.G., Johnson R.A., Wadley H.N.G. (1997). A Monte Carlo simulation of the physical vapor deposition of nickel. Acta Mater..

[31] Lu Y., Wang C., Gao Y., Shi R., Liu X., Wang Y. (2012). Microstructure map for self organized phase separation during film deposition. Phys. Rev. Lett..

[32] Wang W.H., Ren X. (2006). Flux growth of high-quality CoFe_2_O_4_ single crystals and their characterization. J. Cryst. Growth.

[33] Moussy J.B., Gota S., Bataille A., Guittet M.J., Guatier-Soyer M., Delille F., Dieny B., Ott F., Doan T.D., Warin P. (2004). Thickness dependence of anomalous magnetic behavior in epitaxial Fe_3_O_4_(111) thin films: Effect of density of antiphase boundaries. Phys Rev. B.

[34] Sawatzky G.A., Van Der Woude F., Morrish A.H. (1969). Mossbauer study of several ferromagnetic spinel. Phys. Rev..

[35] Murray P.J., Linnett J.W. (1976). Mössbauer studies in the spinel system Co_x_Fe_3-x_O_4_. J. Phys. Chem. Solids.

[36] Skomski R. (2008). Simple Models for Magnetism.

[37] Herklotz A., Gai Z., Sharma Y., Huon A., Rus S.F., Sun L., Shen J., Rack P.D., Ward T.Z. (2018). Designing magnetic anisotropy through strain doping. Adv. Sci..

[38] Bozorth R.M., Walker J.G. (1952). Magnetostriction of single crystals of cobalt and nickel ferrites. Phys. Rev..

[39] Aimon N.M., Hun Kim D., Kyoon Choi H., Ross C.A. (2012). Deposition of epitaxial BiFeO_3_/CoFe_2_O_4_ nanocomposites on (001) SrTiO_3_ by combinatorial pulsed laser deposition. Appl. Phys. Lett..

[40] Callen E.R., Callen H.B. (1963). Magnetoelastic Coupling in Cubic Crystal. Phys. Rev..

