# Supplementary material for: Vertically Aligned Single-Crystalline CoFe2O4 Nanobrush Architectures with High Magnetization and Tailored Magnetic Anisotropy
Source: Nanomaterials (Basel). 2020 Mar 5;10(3):472. doi: 10.3390/nano10030472 (PMC7153250; doi:10.3390/nano10030472)
Supplement: Supplementary file 1 [file nanomaterials-10-00472-s001.pdf]

Supplementary Information

Vertically Aligned Single-Crystalline $CoFe_2O_4$ Nanobrush Architectures with High Magnetization and Tailored Magnetic Anisotropy

Lisha Fan [1,2,†], Xiang Gao [1,†,#], Thomas O. Farmer [1], Dongkyu Lee [1], Er-Jia Guo [1], Sai Mu [1], Kai Wang [1], Michael R. Fitzsimmons [1,3], Matthew F. Chisholm [1], Thomas Z. Ward [1], Gyula Eres [1] and Ho Nyung Lee [1,*]

[1] Oak Ridge National Laboratory, Oak Ridge, TN 37831, USA; lfan@zjut.edu.cn (L.F.); g.xiang@outlook.com (X.G.); thomas.farmer27@hotmail.co.uk (T.O.F.); dongkyu@cec.sc.edu (D.L.); ejguo@iphy.ac.cn (E.-J.G.); sai.mu1986321@gmail.com (S.M.); wangkai.mse@outlook.com (K.W.); fitzsimmonsm@ornl.gov (M.R.F.); chisholmmf@ornl.gov (M.F.C.); wardtz@ornl.gov (T.Z.W.); eresg@ornl.gov (C.E.)

[2] College of Mechanical Engineering, Zhejiang University of Technology, Hangzhou, Zhejiang, 310023, China

[3] Department of Physics and Astronomy, University of Tennessee, Knoxville, TN 37996, USA

[#] Current address: Center for High Pressure Science and Technology Advanced Research, Beijing 100094, China

[*] Correspondence: hnlee@ornl.gov

[†] These authors contributed equally to this work

1. X-ray Diffraction (XRD) of the $CoFe_2O_4$ (CFO) Samples Prepared as Functions of Kinetic Growth Parameters

CFO samples were prepared using pulsed laser epitaxy at a fixed number of pulse (37500 pulses) and a fixed laser repetition rate (15 Hz), as functions of oxygen pressure, $p(O_2)$, from 0.1 to 1 Torr, substrate temperature, T, from 400 to 700 °C, and laser fluence, J, from 1.2 to 2.1 J/cm^2. The XRD θ–2θ scans in Figure S1 show that all CFO samples were epitaxially grown on $SrTiO_3$ (STO) (001) with only 00l series of CFO peaks, except for the one prepared at 1 Torr that shows weak lll peaks, sign of impure orientation.

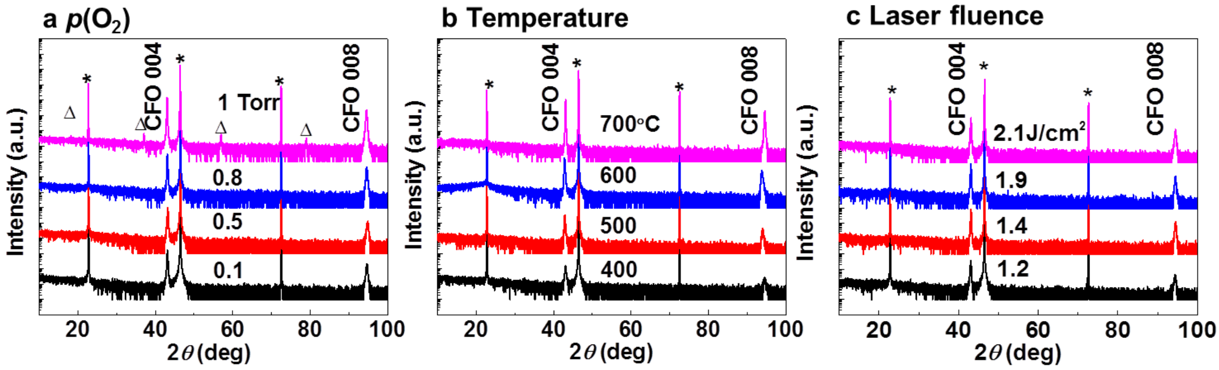

Figure S1. XRD θ–2θ scans of $CoFe_2O_4$ (CFO) samples prepared at different (**a**) $p(O_2)$, (**b**) substrate temperatures, T, and (**c**) laser fluences, J. Other growth conditions for samples shown in (a) are T = 700 °C and J = 1.9 J/cm^2; for (b), $p(O_2)$ = 0.8 Torr and J = 1.9 J/cm^2; for (c), $p(O_2)$ = 0.8 Torr and T = 700 °C. * denotes STO 00l reflections and Δ denotes CFO lll reflections.

2. The Crystallinity of The Micrometer-Tall Sample NB1100

The crystallinity of the micrometer-tall Sample NB1100 was evaluated by full XRD characterization (Figure S2). The typical full width at half maximum (FWHM) of the CFO 004 reflection (Figure S2a) is ~0.38°, revealing a good epitaxial quality. The in-plane ϕ scans (Figure S2b)

of the CFO 101 and STO 101 reflections at ψ = 45°, reveal a clear four-fold symmetry. The results indicate that the sample consists of cube-on-cube epitaxial CFO grown on STO (001).

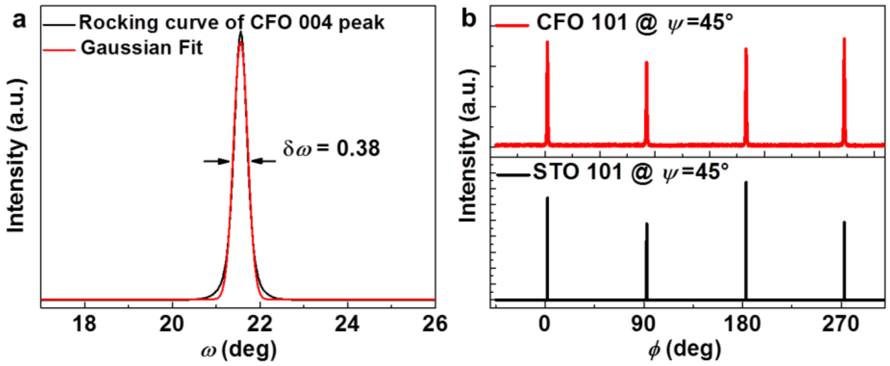

Figure S2. (**a**) An XRD rocking curve of CFO 004 peak and (**b**) in-plane ϕ scans of CFO 101 and STO 101 reflections at ψ = 45° of the micrometer-tall Sample NB1100.

3. Morphology and Structural Characterization of Nanobrush Samples and a 2D Continuous Film

Figure S3 shows the morphology and structural characterization of the nanobrush samples and the 2D continuous film, of which the magnetic properties were compared in the manuscript. The 1100 nm tall nanobrush Sample NB1100 shows loosely packed pyramid heads while the 70 nm tall Sample NB70 prepared at the nanobrush-forming condition has a rough patterned surface morphology, suggesting the initial formation of the nanobrush root. The 180 nm thick 2D continuous film (2DCF) was prepared at a different condition: a lower $p(O_2)$ of 10 mTorr, a substrate temperature of 700 °C and a smaller laser fluence of 0.8 J/cm^2. It has a flat surface, suggesting continuous dense film growth. The XRD θ–2θ scans of these films show that all the films were epitaxially grown on STO (001) with pure 00*l* peaks. The FWHM values of the CFO 004 peak rocking curves of NB1100 and NB70 are 0.38° and 0.73°, respectively, suggesting that the crystallinity is improved as the nanobrush portion in the film increases. The FWHM value of the 2DCF is 0.83°, more than twice higher than that of NB1100, suggesting a lower crystalline quality. The high crystallinity of NB1100 matches the STEM observation that each nanobrush is an almost defect-free single crystal.

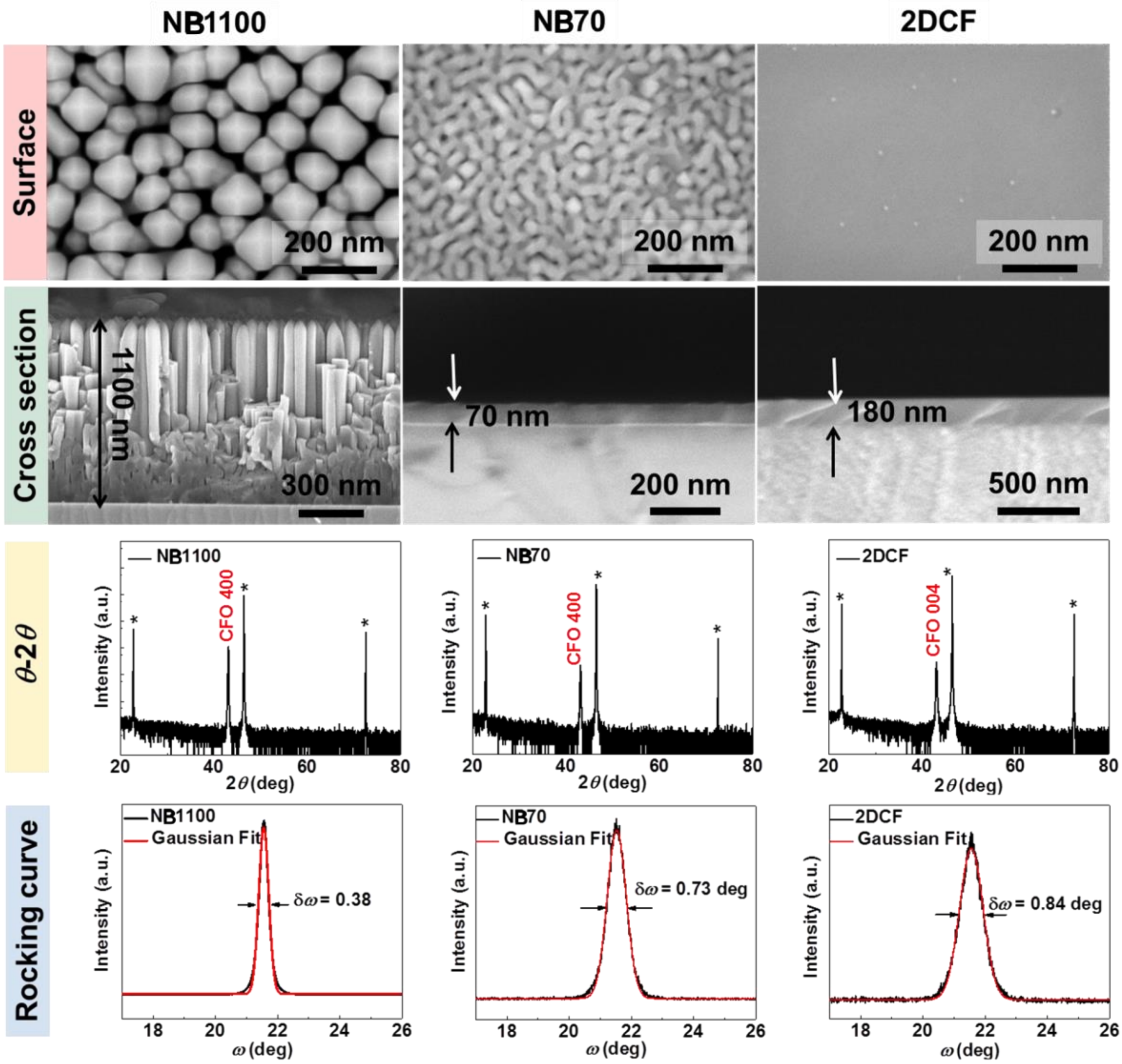

Figure S3. Plan-view SEM images, cross-sectional SEM images, XRD θ–2θ scans, and CFO 004-reflection rocking curves of the NB1100, NB70, and 2DCF, respectively.

4. Estimation of Volume Fraction of Sample NB1100

The volume fraction of Sample NB1100 was estimated by processing the plan-view SEM image (Figure S4a) using a Prewitt filter for edge detection (Figure S4b). A search routine with an absolute tolerance was used to fill the pillars. The porosity of nanobrushes excluding (including) the edges, as shown in Figures S4c,d, was used as an upper (lower) bound. This resulted in a porosity of 0.37 ± 0.1 and a volume fraction of 0.63 ± 0.1 for NB1100. The distribution of the nanobrush cross-sectional areas is shown in Figure S4e. Although the sample size is relatively small, it appears that there are two distributions: large nanobrushes (~7000 nm^2) in which a second distribution of small nanobrushes fills the space, significantly increasing the volume fraction.

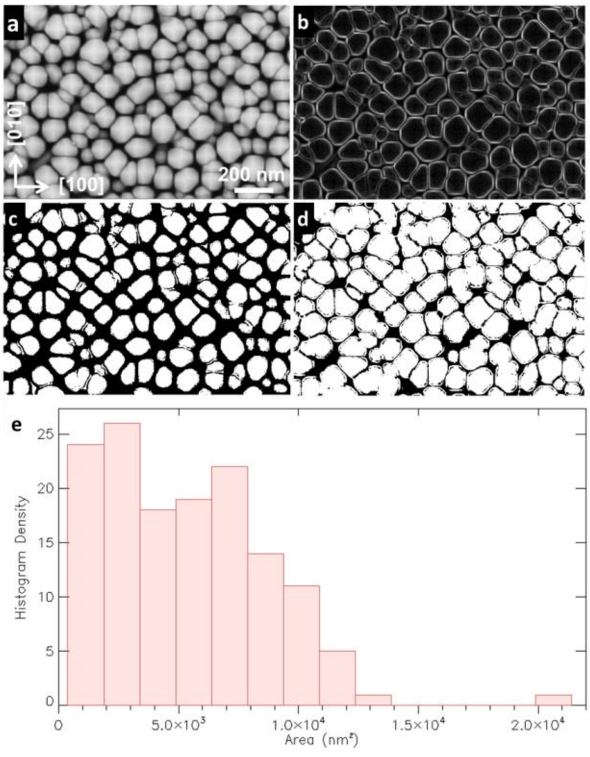

Figure S4. (**a**) SEM image of surface morphology of the NB1100, (**b**) edge detection using Prewitt filter, (**c**) fill of all internal areas in (**b**) with edges excluded in final result, (**d**) fill of all internal areas in (**b**) with edges included in final result, and (**e**) histogram of CFO nanobrush areas.

5. Interpretation of $M_s(T)$ and $H_c(T)$ Data

According to Callen-Callen rule, temperature dependent magnetic anisotropy, which scales $H_c(T)$ in the Stoner-Wohlfarth model, follows a power law of magnetization: $M(T)^n$, where $n = m(m + 1)/2$ for crystal structure with m^{th} order anisotropy. For cubic CFO, $m = 4$ and $n = 10$. We measured temperature-dependent M-H curves of the NB1100 as shown in Figure S5a and retrieved the $H_c(T)$ and $M_s(T)$ from them. The $H_c(T)$ and $M_s(T)$ were normalized by dividing them by $H_c(0)$ and $M_s(0)$, respectively. $H_c(T)/H_c(0)$ and $(M_s(T)/M_s(0))^{10}$ were plotted in Figure S5b. From the scaling as shown in Figure S5b, we observed a much faster $H_c(T)/H_c(0)$ reduction than n = 10 in $(M_s(T)/M_s(0))^n$.

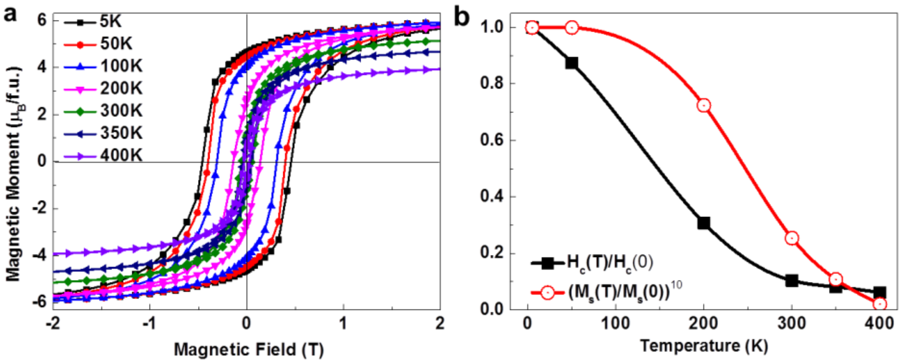

Figure S5. (**a**) Temperature-dependent M-H curves of Sample NB1100. (**b**) Plots of $H_c(T)/H_c(0)$ and $(M_s(T)/M_s(0))^{10}$ as a function of temperature.
